# Dietary Inorganic Nitrate as an Ergogenic Aid: An Expert Consensus Derived via the Modified Delphi Technique

**DOI:** 10.1007/s40279-022-01701-3

**Published:** 2022-05-23

**Authors:** Oliver M. Shannon, Jason D. Allen, Raul Bescos, Louise Burke, Tom Clifford, Chris Easton, Javier T. Gonzalez, Andrew M. Jones, Kristin L. Jonvik, Filip J. Larsen, Peter Peeling, Barbora Piknova, Mario Siervo, Anni Vanhatalo, Kerry McGawley, Simone Porcelli

**Affiliations:** 1 Human Nutrition Research Centre, Population Health Sciences Institute, Newcastle University, Newcastle Upon Tyne, UK; 2 Department of Kinesiology, School of Education and Human Development and Division of Cardiovascular Medicine, School of Medicine, University of Virginia, Charlottesville, VA USA; 3 School of Health Professions, Faculty of Health, Plymouth Institute of Health and Care Research (PIHR), University of Plymouth, Plymouth, UK; 4 Mary MacKillop Institute for Health Research, Australian Catholic University, Melbourne, VIC Australia; 5 School of Sport, Exercise and Health Sciences, Loughborough University, Loughborough, UK; 6 Institute for Clinical Exercise and Health Sciences, University of the West of Scotland, Blantyre, UK; 7 Department for Health, University of Bath, Bath, UK; 8 Centre for Nutrition and Exercise Metabolism, University of Bath, Bath, UK; 9 Sport and Health Sciences, University of Exeter, St Luke’s Campus, Heavitree Road, Exeter, UK; 10 Department of Physical Performance, Norwegian School of Sport Sciences, Oslo, Norway; 11 Department of Physiology, Nutrition and Biomechanics, Åstrand Laboratory, The Swedish School of Sport and Health Sciences, Stockholm, Sweden; 12 School of Human Sciences (Exercise and Sport Science), The University of Western Australia, Crawley, WA Australia; 13 Molecular Medicine Branch, NIDDK, NIH, Bethesda, MD USA; 14 School of Life Sciences, The University of Nottingham Medical School, Queen’s Medical Centre, Nottingham, UK; 15 Swedish Winter Sports Research Centre, Department of Health Sciences, Mid Sweden University, Östersund, Sweden; 16 Department of Molecular Medicine, University of Pavia, Pavia, Italy

## Abstract

**Introduction:**

Dietary inorganic nitrate is a popular nutritional supplement, which increases nitric oxide bioavailability and may improve exercise performance. Despite over a decade of research into the effects of dietary nitrate supplementation during exercise there is currently no expert consensus on how, when and for whom this compound could be recommended as an ergogenic aid. Moreover, there is no consensus on the safe administration of dietary nitrate as an ergogenic aid. This study aimed to address these research gaps.

**Methods:**

The modified Delphi technique was used to establish the views of 12 expert panel members on the use of dietary nitrate as an ergogenic aid. Over three iterative rounds (two via questionnaire and one via videoconferencing), the expert panel members voted on 222 statements relating to dietary nitrate as an ergogenic aid. Consensus was reached when > 80% of the panel provided the same answer (i.e. yes or no). Statements for which > 80% of the panel cast a vote of insufficient evidence were categorised as such and removed from further voting. These statements were subsequently used to identify directions for future research.

**Results:**

The 12 panel members contributed to voting in all three rounds. A total of 39 statements (17.6%) reached consensus across the three rounds (20 yes, 19 no). In round one, 21 statements reached consensus (11 yes, 10 no). In round two, seven further statements reached consensus (4 yes, 3 no). In round three, an additional 11 statements reached consensus (5 yes, 6 no). The panel agreed that there was insufficient evidence for 134 (60.4%) of the statements, and were unable to agree on the outcome of the remaining statements.

**Conclusions:**

This study provides information on the current expert consensus on dietary nitrate, which may be of value to athletes, coaches, practitioners and researchers. The effects of dietary nitrate appear to be diminished in individuals with a higher aerobic fitness (peak oxygen consumption [*V̇*O_2peak_] > 60 ml/kg/min), and therefore, aerobic fitness should be taken into account when considering use of dietary nitrate as an ergogenic aid. It is recommended that athletes looking to benefit from dietary nitrate supplementation should consume 8–16 mmol nitrate acutely or 4–16 mmol/day nitrate chronically (with the final dose ingested 2–4 h pre-exercise) to maximise ergogenic effects, taking into consideration that, from a safety perspective, athletes may be best advised to increase their intake of nitrate via vegetables and vegetable juices. Acute nitrate supplementation up to ~ 16 mmol is believed to be safe, although the safety of chronic nitrate supplementation requires further investigation. The expert panel agreed that there was insufficient evidence for most of the appraised statements, highlighting the need for future research in this area.

**Graphical Abstract:**

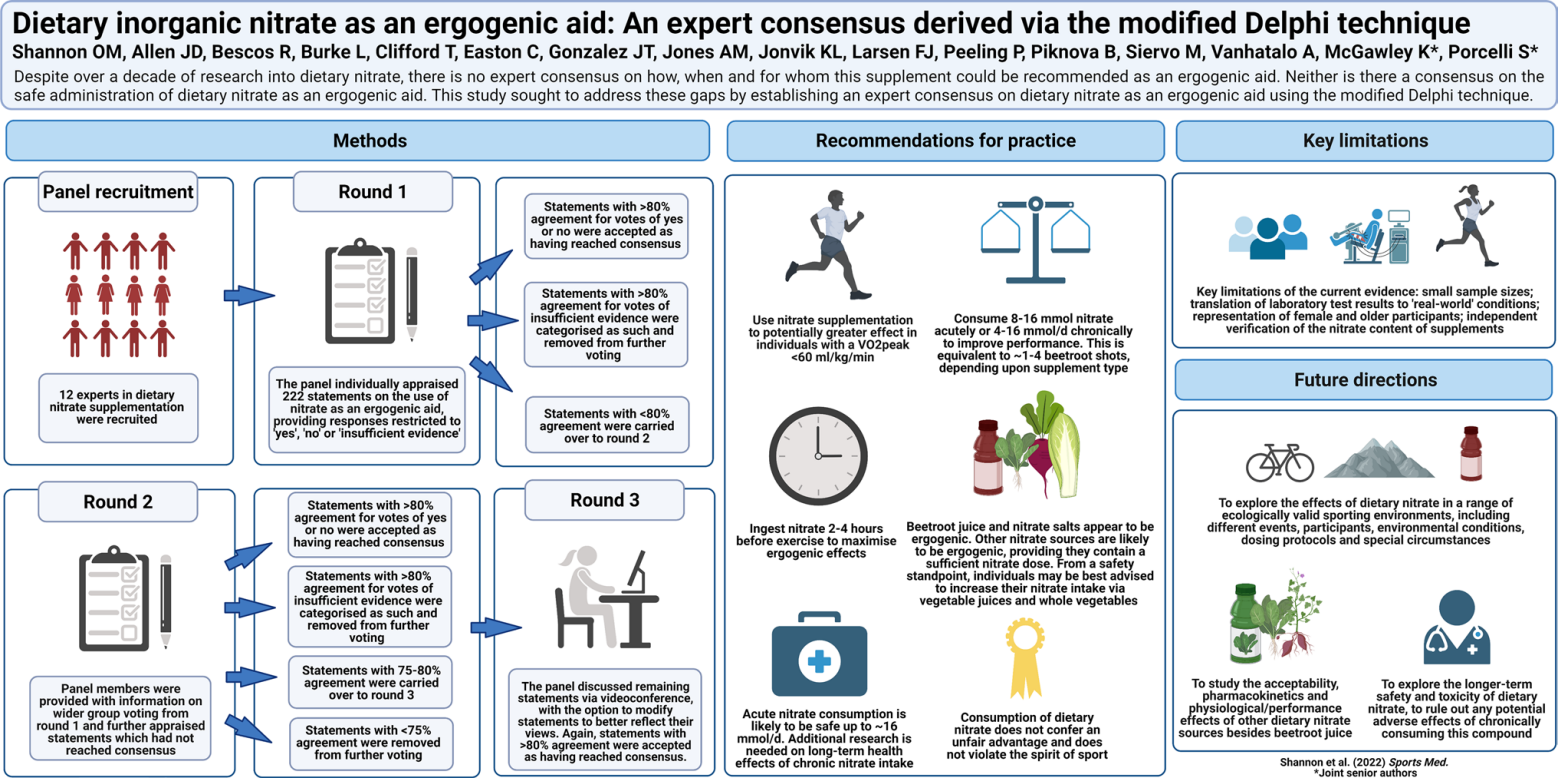

**Supplementary Information:**

The online version contains supplementary material available at 10.1007/s40279-022-01701-3.

## Key Points

**Table:** 

Dietary nitrate is a popular nutritional supplement, yet there is currently no expert consensus on how, when and for whom this compound could be recommended as an ergogenic aid.
This expert consensus provides important information on potential modifiers of the ergogenic effects of dietary nitrate, including details about which supplementation strategies are likely to be efficacious, and which populations are likely to benefit from dietary nitrate supplementation.
Expert judgements concerning the safety and toxicity of dietary nitrate consumption are provided, which could help athletes and coaches weigh up the potential advantages and disadvantages of nitrate supplementation.

## Introduction

Inorganic nitrate is a naturally occurring dietary compound that is mainly found in vegetables and has emerged as a promising ergogenic aid in recent years [1, 2]. Although inorganic nitrate itself is relatively inert, metabolic pathways have been identified in humans that convert this compound into bioactive nitrite and nitric oxide (NO), which have the potential to elicit a wide range of physiological effects [3, 4]. Research from several independent groups has shown that both acute and chronic consumption of inorganic nitrate, which increases NO bioavailability, can improve performance across various time-trial [5–9], time-to-exhaustion [10, 11], high-intensity intermittent [12, 13] and strength-based [14, 15] exercise tasks, as well as exercise tolerance in certain clinical populations [16]. However, dietary nitrate ingestion does not enhance exercise performance under all conditions (e.g. [17–22]), and the different effects of nitrate reported between studies could be related to the nature of the exercise test/protocol, participant characteristics and supplementation strategy, amongst other factors [23–25]. Identifying the specific situations where dietary nitrate is likely to be ergogenic is a topic of considerable interest.

Although dietary nitrate is a popular nutritional supplement among athletes [24], the acceptable daily intake (ADI) for this compound (i.e. the maximum amount that can be ingested daily over a lifetime without increasing risk of adverse events) remains at a relatively low 3.7 mg/kg/day [26], which can easily be exceeded by consumption of a vegetable-rich diet or ingestion of high-nitrate supplements, as is common among athletes [27]. For example, two beetroot juice shots containing a total of 800 mg of inorganic nitrate would provide over three times the ADI of 259 mg/day for a 70-kg individual. The ADI restricting dietary nitrate intake was originally based on observational and animal model data, which suggested that acute or chronic consumption of nitrate could increase the risk of methaemoglobinaemia, whilst chronic nitrate intake could increase risk of cancer [27, 28]. Methaemoglobinaemia is a rare condition in which the ferrous iron (Fe^2+^) in haemoglobin is oxidized into the ferric (Fe^3+^) state, forming methaemoglobin [29]. This condition is typically caused following ingestion/inhalation of an oxidizing agent [30], and recent research has suggested that inorganic nitrate alone does not cause methaemoglobinaemia [27]. In addition, the notion that chronic nitrate intake increases the risk of cancer—an effect which could be caused by increased formation of potentially carcinogenic *N*-nitroso compounds [27]—has been refuted by some [31], but not all [32, 33], recent studies. Meanwhile, reviews by both the International Agency for Research on Cancer (IARC) [34] and the World Health Organisation (WHO) [35] have concluded that there is insufficient evidence for an association between nitrate intake and cancer risk. Recently, it has been suggested that dietary nitrate may elicit certain beneficial health effects, including reduced cardiovascular disease risk factors [36–39]. Moreover, a dose–response relationship has been reported with dietary nitrate, with moderate-to-high doses eliciting greater ergogenic effects, and more pronounced effects on parameters such as blood pressure, compared with lower doses [40]. Further consideration of the dietary nitrate ADI, and its applicability for individuals taking part in sport and exercise, may therefore be warranted.

Despite accumulating evidence for the potential ergogenic effects of inorganic nitrate under certain circumstances, including reviews by independent leaders in the field [1, 41, 42], there is a lack of expert consensus focusing specifically on how, when and for whom dietary nitrate could be recommended as an ergogenic aid. Similarly, there is currently no consensus on the specific situations in which, and individuals for whom, nitrate may not be efficacious, yet such knowledge could be valuable to help avoid ineffective dietary supplementation with this compound. In addition, there is no consensus on the safe administration of dietary nitrate as an ergogenic aid. An expert consensus statement would be valuable to provide guidelines that may be used to inform athlete, coach and practitioner decision making. Additionally, by synthesising the views of experts from within the field, it is possible to identify key areas of ambiguity and focus attention on potential target areas for future research, which can benefit both research and sporting communities.

Consensus from research scientists can be established via various methodological approaches [43], including the Delphi technique, which is a scientific approach for generating a consensus on the current state of knowledge on any given topic [44]. The Delphi technique has previously been employed in various areas of sport and exercise science and specifically in determining best practice for the administration of different nutritional supplements and ergogenic aids [45, 46]. The current study aims to use this technique to derive a consensus on the use of dietary inorganic nitrate as an ergogenic aid, focusing on seven key areas of interest, including the following: (1) identifying the specific types of sport/exercise for which dietary nitrate is ergogenic (*Activity*); (2) characterizing the specific populations for whom dietary nitrate is ergogenic (*Population*); (3) identifying which dietary nitrate supplementation strategies are ergogenic (*Supplementation strategy*); (4) elucidating the physiological changes that underpin the ergogenic effects of dietary nitrate (*Physiological effects*); (5) clarifying whether dietary nitrate is safe to consume (*Safety and toxicity*); (6) appraising the quality of the evidence for dietary nitrate as an ergogenic aid (*Quality of available evidence*); (7) determining whether supplementation with dietary inorganic nitrate for ergogenic purposes is consistent with the ethos of Olympic sport (*Ethos of Olympic sport*).

## Methods

### Overview

This study, which was approved by the Newcastle University Ethics Committee (9226/2020), used the modified Delphi technique to derive a consensus on the use of dietary inorganic nitrate as an ergogenic aid. Similar to previous research [45], the core research team (OMS, KM and SP) developed a series of statements that focused on the aforementioned seven key areas relating to the use of dietary inorganic nitrate as an ergogenic aid (Activity, Population, Supplementation strategy, Physiological effects, Safety and toxicity, Quality of available evidence and Ethos of Olympic sport). Twelve expert panel members were then invited to vote on the final 222 statements over three iterative rounds, between February and July, 2021. The first two rounds were conducted anonymously, with each panel member providing votes via a custom-made, electronic questionnaire. The third round involved face-to-face discussion via videoconferencing software. This allowed panel members to discuss specific statements that lacked consensus, to provide explanations for their voting and to modify or add a caveat to statements to better reflect the group’s collective views.

### Panel Selection

To be eligible as a panel member, prospective experts were required to possess a PhD in sports/exercise/nutrition science or a related discipline and fit at least one of the following criteria:

- One or more first or senior author publications examining the ergogenic effect of dietary inorganic nitrate
- One or more first or senior author publications examining the physiology/metabolism of dietary inorganic nitrate
- An applied practitioner with practical experience using dietary inorganic nitrate as an ergogenic aid with athletes or clinical populations

The following procedures were adhered to when selecting the expert panel: (1) initially contacting individuals who were perceived to have the greatest expertise in the area; (2) recruiting no more than two individuals from the same institution to minimise skewing the overall consensus towards the views of one specific working group; (3) recruiting individuals from a number of countries to reflect international opinion; (4) having a balance between applied and mechanistic expertise; and (5) ensuring representation of both men and women on the panel. The core research team created a list of potential panel members and initially contacted 12 of these experts to ensure a final sample of at least ten panel members, which is consistent with previous research [45] and is deemed to be sufficient to make up a consensus group [47]. Of the first 12 experts initially contacted, one declined on account of a perceived conflict of interest, so a 13th was subsequently selected. The final 12 expert panel members all provided fully informed written consent prior to receiving the initial questionnaire. The panel does not represent an exhaustive list of experts in the area, but instead reflects a cross-section of significant contributors to the field from scientific and practitioner perspectives.

### Questionnaire Development

A questionnaire was developed by the core research team and included 222 statements across the seven key areas relating to the use of dietary inorganic nitrate as an ergogenic aid. An initial list of statements was compiled following a review of the extant literature, with a particular focus on the findings from recently published review articles (e.g. [1, 2, 48, 49]). The statements were further modified by the core research team based on their practical experience gained through dietary nitrate use with athletes and clinical populations in laboratory and applied settings. Further iterations of the process allowed the core research team to identify potential omissions or issues with clarity, until the final list was agreed upon. As suggested by Hasson et al. [50], pilot testing was conducted to help refine the questionnaire prior to implementation. Here, an independent, impartial researcher with expertise in dietary nitrate supplementation provided feedback on the structure and clarity of the statements, and the questionnaire was modified accordingly [51].

### Round One

**Fig. 1:**
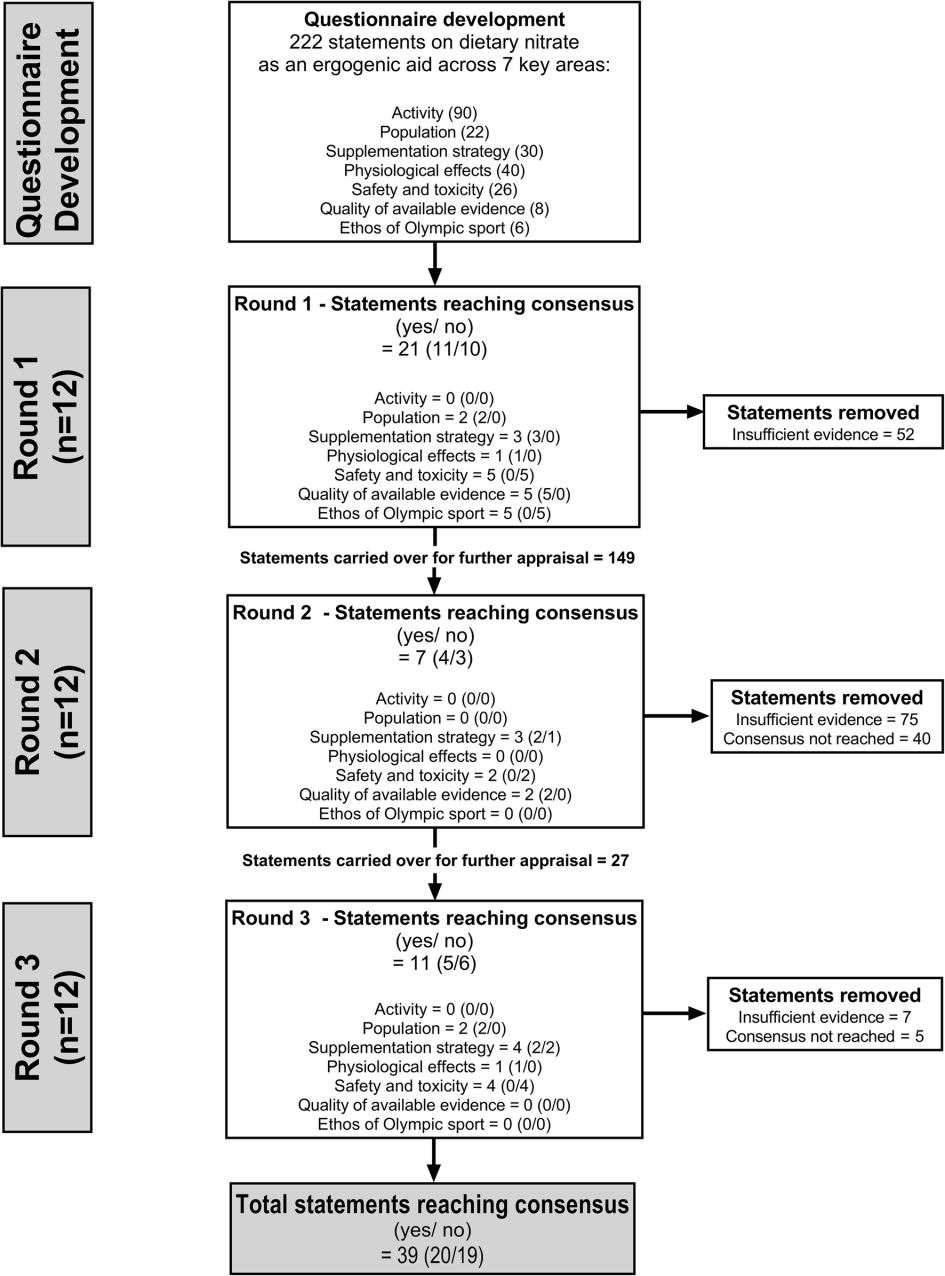
Flow chart showing statements accepted as having reached consensus in/across each round of the study. Numbers in parentheses for rounds 1, 2 and 3 reflect the number of statements reaching consensus under the headings of yes/no

In round one of the data collection, panel members were provided with a spreadsheet containing the 222 statements relating to the use of dietary inorganic nitrate as an ergogenic aid. Instructions were provided on how to complete the spreadsheet, and clarification was provided by the core research team where required. All panel members, including those from the same research group, were asked to complete the spreadsheet independently. Panel members were asked to critically appraise each statement and then vote by selecting ‘yes’, ‘no’, or ‘insufficient evidence’ in a designated voting column. The insufficient evidence option was provided for cases where panel members felt that there was insufficient evidence to conclusively state yes or no, or if they felt that their own knowledge was insufficient to respond to the statement. Panel members were asked to complete the round one spreadsheet and return it to the core research team within 2 weeks, after which the responses were collated. In accordance with previous research [45, 47, 52], statements were accepted as having reached consensus when the same response (i.e. yes or no) was provided by > 80% (ten or more) of the panel. These statements provide insight into what is currently known about dietary nitrate as an ergogenic aid by experts in the field. Statements for which > 80% of the panel cast a vote of insufficient evidence were categorised as such and removed from further voting. Panel members were asked to provide an explanation to clarify why they selected insufficient evidence, using the following three options: (1) a lack of evidence available to support a particular statement; (2) evidence was equivocal; or (3) insufficient knowledge to respond to the statement. These statements provided insight into what is currently unknown about dietary nitrate as an ergogenic aid by experts in the field and were used to identify directions for future research. The cut-off at which consensus was deemed to have been achieved was defined a priori to minimise bias [44]. Statements that had not reached consensus or had not been removed from voting due to insufficient evidence in round one were carried over to round two for further consideration by the panel (Fig. 1).

### Round Two

In round two of the data collection, panel members were provided with a summary of the statements that had reached consensus in round one, together with a new spreadsheet containing all remaining statements. Next to each statement on the new spreadsheet, panel members were provided with information on their own vote from round one and an anonymous summary of the wider group voting. Panel members were asked to carefully consider their vote from the previous round and, reflecting on the wider group voting, decide whether they wished to support (i.e. cast the same vote again) or change their response. The same voting options of yes, no, or insufficient evidence were available in this round. Panel members were also asked to provide a brief justification for their response, which could include a short sentence or appropriate reference. Panel members were asked to complete and return round two spreadsheets within 3 weeks, after which the responses were collated. Statements with > 80% agreement (i.e. ten or more of the panel) for votes of yes or no were accepted as having reached consensus. Meanwhile, statements for which > 80% of the panel cast a vote of insufficient evidence were categorised as such and removed from further voting. Again, panel members were asked to clarify their reasons for selecting this option. Statements with ≥ 75% agreement (i.e. nine of the panel), but not yet having passed the 80% threshold, were carried over to round three for further appraisal. All other statements not reaching this 75% threshold were removed from additional voting and categorised as having not reached consensus (Fig. 1). There is no universally accepted threshold for determining consensus or whether to carry over statements to a subsequent round [50]. In this study, the decision to only carry over statements with ≥ 75% agreement to round three was taken following a group discussion between the core research team and moderator (an impartial individual recruited to moderate the discussion in round three, outlined in more detail below), considering the advantages and disadvantages of applying different thresholds for inclusion. The final decision was based on (1) a practical assessment of the time needed for 12 experts to discuss those statements approaching consensus and (2) the assumption that the panel members were unlikely to reach a consensus in round three on statements with a lower level of agreement, even with further discussion [53].

### Round Three

In round three of the data collection, panel members were invited to attend an ~ 2.5-h videoconference call where the remaining statements were discussed. Prior to attending the meeting, panel members were provided with a spreadsheet summarizing all statements accepted until this point, alongside those to be discussed in round three. As per round two, this included information about the individual panel member’s previous vote, together with an anonymous summary of the wider group voting. Panel members were encouraged to consider each remaining statement prior to the videoconferencing call to facilitate efficient discussion in the final round. An independent researcher (JTG) who had not been involved in the previous stages of the project was recruited to moderate the discussion, and to ensure that all panel members had the opportunity to express their opinions. The moderator was provided with the list of statements prior to the discussion, together with potential prompts and relevant background information. They also met with the core research team for ~ 30 min prior to the expert panel discussion to clarify any areas of ambiguity and discuss the practicalities of moderating the group discussion. During the expert discussion, the moderator presented each statement to the panel and facilitated debate. Where appropriate, panel members were able to modify statements or add in a caveat to better reflect the views of the group. Here, the moderator helped synthesise the views of the panel to refine an existing statement, if required. Voting was then cast via the group chat function, with the same voting options available as in previous rounds (i.e. yes, no, or insufficient evidence). Unlike in rounds one and two, voting in round three was no longer anonymous. As per previous rounds, statements with > 80% agreement (i.e. ten or more of the panel) for votes of yes or no were accepted as having reached consensus. Likewise, statements for which > 80% of the panel cast a vote of insufficient evidence were categorised as such and removed from further voting. All other statements were categorised as having not reached consensus (Fig. 1).

## Results

### Expert Participation

The final expert panel included 12 members (eight men and four women; six full professors, three associate professors/senior lecturers, one lecturer, and two researchers/staff scientists) from 11 different institutions and five different countries (the United Kingdom, United States, Australia, Norway and Sweden). All 12 panel members completed the round one and round two questionnaires. Eleven of the 12 panel members attended the videoconference call in round three, although two of these 11 were obliged to leave the meeting after discussing five and 19 (of 27) statements, respectively. To benefit from the expertise of all panel members in forming the final consensus statement, the three individuals not attending the entire meeting were provided with a full recording of the videoconference call and a summary of the group voting from round three. They were then asked to cast their votes, whilst considering arguments and votes presented by the wider panel during the videoconference call. The final consensus statement, therefore, reflects the overall group votes of 12 expert panel members (i.e. 100% retention).

### Voting Summary

**Fig. 2:**
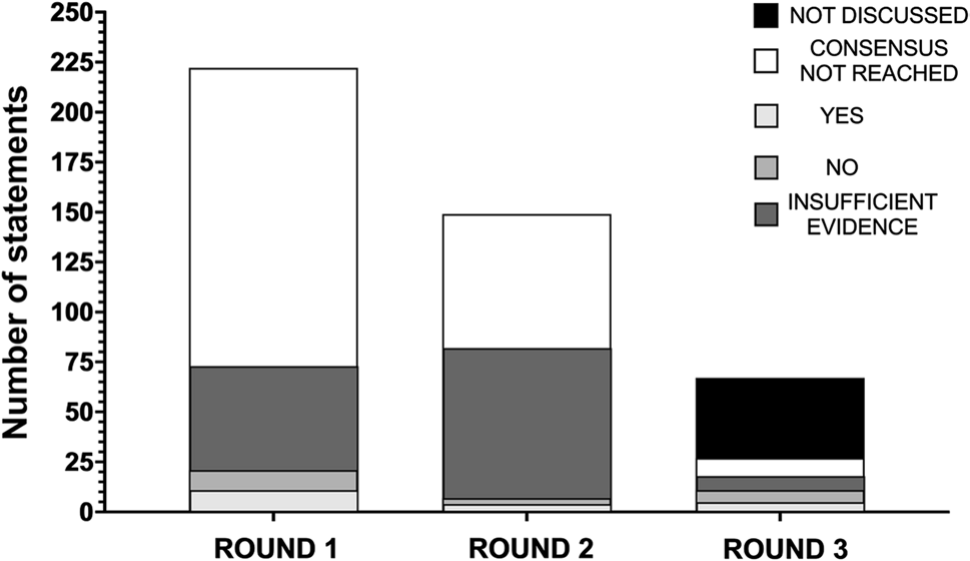
A summary of overall voting across each of the three rounds. Statements are broken down into those for which consensus was not reached (white), those for which it was reached for yes (light grey) and no (medium grey), and those for which the panel agreed there was insufficient evidence (dark grey). For round 3, statements not discussed are also presented (black)

In total, a consensus was reached for 39 (17.6%) of the statements (20 yes, 19 no). The panel also agreed that there was insufficient evidence for 134 (60.4%) of the statements. The insufficient evidence option was primarily selected when panel members felt there was a lack of evidence available to support a particular statement (82.7% of the time), rather than when they felt evidence was equivocal (7.6% of the time) or when they believed they had insufficient knowledge to respond to the statement (9.7% of the time). A summary of the voting across the three rounds is displayed in Fig. 2 and is presented in more detail in the Electronic Supplementary Material.

#### Round One

**Fig. 3:**
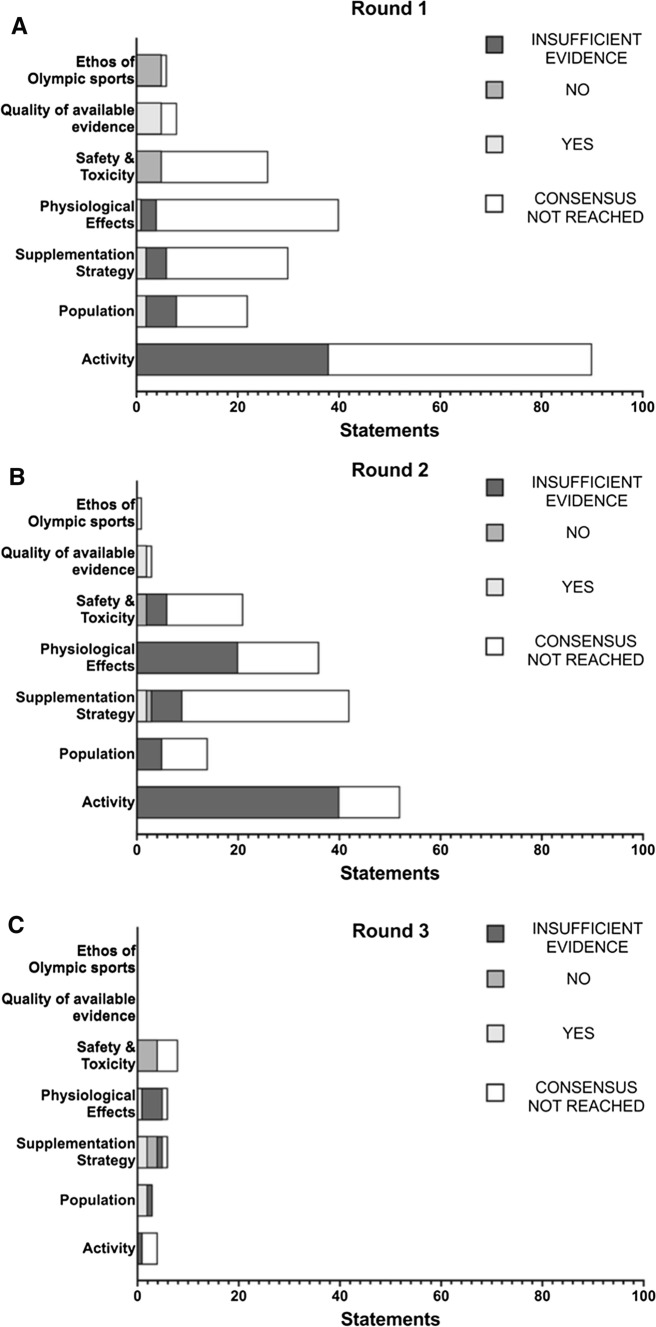
A summary of votes cast in each of the seven key areas of interest in round 1 (**A**), round 2 (**B**) and round 3 (**C**). Statements are broken down into those for which consensus was not reached (white), those it was reached for yes (light grey) and no (medium grey), and those for which the panel agreed there was insufficient evidence (dark grey)

In round one, a total of 21 of 222 statements (11 yes, 10 no; Tables 1 and 2) reached consensus, whilst 52 statements were removed from further voting due to insufficient evidence. As shown in Fig. 3, this included the following number of statements reaching consensus for each of the review sections: Activity = 0/90 (0 yes, 0 no), Population = 2/22 (2 yes, 0 no), Supplementation strategy = 3/30 (3 yes, 0 no), Physiological effects = 1/40 (1 yes, 0 no), Safety and toxicity = 5/26 (0 yes, 5 no), Quality of available evidence = 5/8 (5 yes, 0 no) and Ethos of Olympic sport = 5/6 (0 yes, 5 no).

**Table 1:** Statements for which a consensus was reached as ‘yes’ (which only occurred under the categories of Population, Supplementation strategy, Physiological effects and Quality of available evidence)

Statement number	Sub-domain	Statement	Yes/no	Round achieved	Modification/caveat
*Population*					
46(a)	Aerobic fitness	Dietary nitrate is ergogenic in individuals with a V̇O_2peak_ of < 45 ml/kg/min—acute supplementation (i.e. administration of a single, one-off nitrate supplement)	Yes	1	–
46(b)	Aerobic fitness	Dietary nitrate is ergogenic in individuals with a V̇O_2peak_ of < 45 ml/kg/min—chronic supplementation (i.e. administration of nitrate over several days/weeks)	Yes	1	–
47(b)	Aerobic fitness	Dietary nitrate is ergogenic in individuals with a V̇O_2peak_ of 45–60 ml/kg/min – chronic supplementation (i.e. administration of nitrate over several days/weeks)	Yes	3	–
55(b)	Health status	Dietary nitrate is ergogenic in healthy individuals; chronic supplementation (i.e. administration of nitrate over several days/weeks)	Yes	3	–
*Supplementation strategy*					
60(c)	Dose	Acute supplementation (i.e. administration of a single, one-off nitrate supplement) is ergogenic for TT and TTE performance in the following doses: 8–16 mmol (496–992 mg)	Yes	3	Statement modified to include TT and TTE tests, as supportive evidence comes from both testing modalities
61(b)	Dose	Chronic supplementation (i.e. administration of nitrate over several days/weeks) is ergogenic in the following doses: 4–8 mmol (248–496 mg)	Yes	1	–
61(c)	Dose	Chronic supplementation (i.e. administration of nitrate over several days/weeks) is ergogenic in the following doses: 8–16 mmol (496–992 mg)	Yes	1	–
61(d)	Dose	Chronic supplementation (i.e. administration of nitrate over several days/weeks) is ergogenic in the following doses: > 16 mmol (> 992 mg)	Yes	2	–
68(d)	Timing	Acute supplementation (i.e. administration of a single, one-off nitrate supplement) is ergogenic when provided 2–4 h pre-exercise	Yes	2	–
71(a)	Form	Dietary nitrate is ergogenic when provided in the following forms: nitrate salts (e.g. sodium or potassium nitrate)	Yes	3	–
71(b)	Form	Dietary nitrate is ergogenic when provided in the following forms: beetroot juice	Yes	1	–
*Physiological effects*					
74(b)	V̇O_2_	Dietary nitrate supplementation reduces V̇O_2_ during steady-state exercise—chronic supplementation (i.e. administration of nitrate over several days/weeks)	Yes	1	–
89(b)	Microbiota	Dietary nitrate supplementation modulates the oral microbiota—chronic supplementation (i.e. administration of nitrate over several days/weeks)	Yes	3	–
*Quality of available evidence*					
105	Study design	The ergogenic effects of nitrate have mostly been established in randomized controlled trials in a laboratory setting using a within-subject design	Yes	1	–
106	Control group	The ergogenic effects of nitrate have mostly been established in studies with an appropriate control group	Yes	1	–
107	Blinding	The ergogenic effects of nitrate have mostly been established in studies using a double-blind design (i.e. participants and researchers blinded to the experimental condition)	Yes	1	–
108	Sample size	The ergogenic effects of nitrate have mostly been established in studies with an inappropriate/insufficient sample size	Yes	1	–
109	Performance test	The ergogenic effects of nitrate have mostly been established in studies with a performance test partly indicative of the conditions that occur in 'real-world' competitive sport	Yes	2	–
110	Verification	The ergogenic effects of nitrate have mostly been established in studies that partly verify that the supplement was taken and induced a biological response (e.g. via saliva, blood, urine or muscle samples)	Yes	2	–
111	Standardisation	The ergogenic effects of nitrate have mostly been established in studies that partly standardise the design to control for confounding variables that may influence the result (e.g. mouthwash use, habitual diet, smoking status, pre-trial exercise levels, sleep, the testing environment)	Yes	1	–

For the statement number, *text in parentheses* is presented when a question contained sub-statements (e.g. separate statements for acute and chronic dosing or different forms, doses, timings of nitrate supplements). When discussing chronic supplementation strategies, the dose refers to the daily intake of inorganic nitrate

*TT* time trial, *TTE* time to exhaustion, *V̇O*_*2*_ oxygen consumption, *V̇O*_*2peak*_ peak V̇O_2_

**Table 2:** Statements for which a consensus was reached as ‘no’ (which only occurred under the categories of Supplementation strategy, Safety and toxicity and Ethos of Olympic sport)

Statement number	Sub-domain	Statement	Yes/no	Round achieved	Modification/caveat
*Supplementation strategy*					
60(a)	Dose	Acute supplementation (i.e. administration of a single, one-off nitrate supplement) is ergogenic in the following doses: < 4 mmol (< 248 mg)	No	2	–
68(b + c)	Timing	Acute supplementation (i.e. administration of a single, one-off nitrate supplement) is ergogenic when provided < 90 min prior to the performance test	No	3	Statement modified from 30 min–2 h to a new time range and to clarify that the timing is with respect to the performance assessment
68(e)	Timing	Acute supplementation (i.e. administration of a single, one-off nitrate supplement) is ergogenic when provided 6–8 h prior to the performance test	No	3	Statement modified from 4 to 8 h to a new time range and to clarify that the timing is with respect to the performance assessment
*Safety and toxicity*					
94(a)	Specific health conditions	Dietary nitrate supplementation increases risk of cancer; acute supplementation (i.e. administration of a single, one-off nitrate supplement)	No	1	–
95(a)	Specific health conditions	Dietary nitrate supplementation increases risk of methaemoglobinaemia—acute supplementation (i.e. administration of a single, one-off nitrate supplement)	No	1	–
95(b)	Specific health conditions	Dietary nitrate supplementation increases risk of methaemoglobinaemia—chronic supplementation (i.e. administration of nitrate over several days/weeks)	No	3	–
96(a)	Specific health conditions	Dietary nitrate supplementation increases risk of hypotension—acute supplementation (i.e. administration of a single, one-off nitrate supplement)	No	1	–
96(b)	Specific health conditions	Dietary nitrate supplementation increases risk of hypotension—chronic supplementation (i.e. administration of nitrate over several days/weeks)	No	2	–
97(a)	Specific health conditions	Dietary nitrate supplementation increases risk of renal injury—acute supplementation (i.e. administration of a single, one-off nitrate supplement)	No	1	–
98	ADI	The ADI of 0–3.7 mg/kg/day nitrate is appropriate based on current evidence	No	2	–
99(a)	Form	Adverse health effects of dietary nitrate supplementation are reported for nitrate-rich foods and juices (e.g. vegetables, salads, concentrated or non-concentrated beetroot juice)	No	1	–
102(a)	Environment	Dietary nitrate increases the risk of heat illness prior to/during exercise in the heat (> 30 °C)—acute supplementation (i.e. administration of a single, one-off nitrate supplement)	No	3	–
102(b)	Environment	Dietary nitrate increases the risk of heat illness prior to/during exercise in the heat (> 30 °C)—chronic supplementation (i.e. administration of nitrate over several days/weeks)	No	3	–
104	Population	Serious adverse health effects of dietary nitrate supplementation have been reported	No	3	Initial statement specified specific population subgroups. These were amalgamated for this final statement. In addition, the term ‘serious’ was added for consistency with typical adverse event reporting in clinical trials
*Ethos of Olympic sport*					
113(a)	Health risk	Dietary nitrate supplementation presents an actual or potential health risk to the athlete/participant—acute supplementation (i.e. administration of a single, one-off nitrate supplement)	No	1	–
114(a)	Spirit of the sport	Dietary nitrate supplementation violates the spirit of the sport—acute supplementation (i.e. administration of a single, one-off nitrate supplement)	No	1	–
114(b)	Spirit of the sport	Dietary nitrate supplementation violates the spirit of the sport—chronic supplementation (i.e. administration of nitrate over several days/weeks)	No	1	–
115(a)	Unfair advantage	Dietary nitrate supplementation gives an unfair advantage to those using it—acute supplementation (i.e. administration of a single, one-off nitrate supplement)	No	1	–
115(b)	Unfair advantage	Dietary nitrate supplementation gives an unfair advantage to those using it—chronic supplementation (i.e. administration of nitrate over several days/weeks)	No	1	–

For the statement number, *text in parentheses* is presented when a question contained sub-statements (e.g. separate statements for acute and chronic dosing or different forms, doses, timings of nitrate supplements)

*ADI* acceptable daily intake

#### Round Two

The remaining 149 statements were entered into round two for reconsideration. A total of seven further statements (4 yes, 3 no; Tables 1 and 2) reached consensus during this round, whilst an additional 75 statements were removed from further voting due to insufficient evidence. As shown in Fig. 3, this included the following number of statements for each of the review sections: Activity = 0/52 (0 yes, 0 no), Population = 0/14 (0 yes, 0 no), Supplementation strategy = 3/42 (2 yes, 1 no), Physiological effects = 0/36 (0 yes, 0 no), Safety and toxicity = 2/21 (0 yes, 2 no), Quality of available evidence = 2/3 (2 yes, 0 no) and Ethos of Olympic sport = 0/1 (0 yes, 0 no).

#### Round Three

Of the 67 remaining statements after round two, 40 were removed prior to the start of round three due to a lack of consistency in group voting (i.e. < 75% agreement). Therefore, 27 statements were discussed by the panel in round three. Eleven statements were modified as part of this round to better reflect the views of the expert panel. This included adjusting the wording of six statements, and collapsing five statements (which discussed the safety of inorganic nitrate for different cohorts) into one unifying statement for all individuals, which reduced the overall number of statements in this round to 23. A total of seven statements were removed from voting due to insufficient evidence, whilst 11 of 23 statements reached consensus during round three (5 yes, 6 no; Tables 1 and 2). As shown in Fig. 3, this included the following number of statements for each of the review sections: Activity = 0/4 (0 yes, 0 no), Population = 2/3 (2 yes, 0 no), Supplementation strategy = 4/6 (2 yes, 2 no), Physiological effects = 1/6 (1 yes, 0 no), Safety and toxicity = 4/8 (0 yes, 4 no). No statements on the Quality of available evidence or Ethos of Olympic sport were discussed in this round.

## Discussion

This study aimed to establish an expert consensus on the use of dietary inorganic nitrate as an ergogenic aid. Over the course of three iterative rounds, 12 expert panel members considered 222 statements across seven distinct areas relating to this topic. A consensus of yes or no was reached for 39 (17.6%) of the statements, whereas the panellists agreed that there was insufficient evidence for 134 (60.4%) of the statements. The findings provide valuable insight into the potential applications of dietary nitrate as an ergogenic aid, highlight areas of ambiguity amongst experts in this area and provide information on potential directions for future research. In the following section, we begin by discussing the statements for which a consensus was reached. We then compare our findings to those of a recent systematic review/meta-analysis, outline strengths and limitations of our study and highlight key recommendations for practice and future research.

### Modulators of the Ergogenic Effects of Dietary Nitrate

#### Population

*Aerobic fitness level:* The expert panel agreed that both acute and chronic dietary nitrate supplementation is ergogenic for individuals with peak oxygen consumption (*V̇*O_2peak_) < 45 ml/kg/min (i.e. low aerobic fitness) and that chronic dietary nitrate supplementation is ergogenic for individuals with a *V̇*O_2peak_ < 60 ml/kg/min (i.e. low and moderate aerobic fitness). This expert view is supported by previous research, which has shown consistent beneficial effects of dietary nitrate (especially when administered chronically) in individuals of lower versus higher aerobic fitness levels [2]. Notably, Porcelli et al. [9] found that 6 days of dietary nitrate supplementation (5.5 mmol/day) improved 3-km running performance in individuals with a low (*V̇*O_2peak_ 28–44 ml/kg/min) and moderate (*V̇*O_2peak_ 46–57 ml/kg/min) aerobic fitness, but not a high (*V̇*O_2peak_ 64–81 ml/kg/min) aerobic fitness. Similarly, the systematic review and meta-analysis by Senefeld et al. [2] revealed an overall ergogenic effect of dietary nitrate among individuals with V̇O_2peak_ values ranging from 40 to 65 ml/kg/min, but not in highly trained athletes with a *V̇*O_2peak_ ≥ 65 ml/kg/min. Some studies have suggested that individual responders to dietary nitrate may exist amongst highly trained athlete populations with a high aerobic fitness [18, 54], although few studies have been appropriately designed to identify responders versus non-responders [55]. As such, further research is needed to clarify whether some highly trained individuals with a high aerobic fitness may benefit from dietary nitrate supplementation and whether responsiveness is a reproducible effect [56]. Interestingly, evidence from Liddle et al. [57] suggests substantial within-person variation in salivary and plasma concentrations of NO biomarkers following dietary nitrate ingestion. Such variation may be related to fluctuations in relative abundances and/or quantities of oral bacteria that are involved in nitrate and/or nitrite reduction. This could translate into different physiological/ergogenic effects of dietary nitrate in the same individual on different occasions.

*Sex:* As highlighted in a recent review by Wickham and Spriet [49], women are underrepresented in research exploring the ergogenic effects of dietary nitrate supplementation. This is consistent with the wider pattern of underrepresentation of women in sport and exercise science research, discussed in detail elsewhere [58–60] and typically explained by the need for additional methodological considerations when carrying out research with female participants (e.g. controlling for sex hormone concentrations, the use of oral contraceptives, impaired menstrual function [59, 61] and nutritional issues such as iron deficiency [62]). Accordingly, the expert panel agreed that there was insufficient evidence to state whether dietary nitrate was more/less effective in men versus women. Interestingly, a small sub-group analysis in the systematic review and meta-analysis of Senefeld et al. [2] found that dietary nitrate did not improve performance in studies containing only women, while Wickham and Spriet [49] have detailed plausible physiological reasons for how the ergogenic effects of dietary nitrate could be attenuated in women. Whilst it is possible that the ergogenic effects of dietary nitrate may be less apparent in women, more research is needed before sex-specific guidelines can be formed.

*Age:* The expert panel agreed that there was insufficient evidence to conclusively state whether the effects of dietary nitrate differ depending upon age. This likely reflects the ambiguity in the current body of evidence, where dietary nitrate has been shown to improve exercise performance in some [16, 63–66] but not all [67–69] studies of older adults (aged > 60 years). Moreover, many studies have included participants with co-morbidities, which makes it difficult to distinguish the effects of age from disease states. Interestingly, Coggan et al. [70] reported similar effects of dietary nitrate on muscle power across a range of ages (22–79 years). However, further research contrasting the effects of dietary nitrate between younger and older adults, and between age-matched healthy and clinical populations, would provide valuable insight into the potential differences in responses to dietary nitrate between these sub-groups.

*Health status:* The expert panel agreed that chronic dietary nitrate supplementation was ergogenic in healthy individuals, but did not reach consensus for acute supplementation being ergogenic in this population. The expert panel also felt that there was insufficient evidence, or were unable to reach consensus, for acute or chronic dietary nitrate supplementation being ergogenic in clinical populations, which is consistent with the mixed findings reported in the current literature for these cohorts (for a review, see [71]). It is important to highlight that only 58% of the expert panel reported having experience of using dietary nitrate with clinical populations, compared with 100% of the panel having experience with healthy or sporting populations. It is therefore possible that some of our panel may not have the necessary expertise to determine whether dietary nitrate is/is not ergogenic in clinical cohorts. Moreover, the panel were presented with relatively few statements relating to the effects of dietary nitrate in clinical populations, compared with the substantial detail used with the sporting categories, and broad definitions were used to categorise clinical populations (e.g. those with cardiovascular, pulmonary and metabolic disease). It is possible that the conclusions may have differed if we had presented the panel with more granular statements relating to individual conditions (e.g. heart failure with preserved ejection fraction vs. heart failure with reduced ejection fraction or peripheral artery disease), which have distinct clinical features, rather than disease types (e.g. cardiovascular disease). Nevertheless, as there was some expertise of using dietary nitrate with clinical populations amongst our panel, and in the interest of presenting a comprehensive view, we decided to include a number of statements relating to the potential ergogenic effects of nitrate depending upon health status. For a comprehensive review of the effects of nitrate in clinical populations, the interested reader is directed to the paper by Woessner et al. [72].

#### Supplementation Strategy

*Dose:* Convincing evidence for the dose-dependent effects of dietary nitrate in healthy, young volunteers has been provided by Wylie et al. [40, 73]. Similarly, in the systematic review and meta-analysis by Senefeld et al. [2], dietary nitrate was suggested to be ergogenic for moderate to high dosages (5.1–28.7 mmol/day), but not for low dosages (1.6–5.0 mmol/day). The expert panel agreed that acute supplementation with a nitrate dose of between 8 and 16 mmol and chronic supplementation with dosages of 4 to > 16 mmol/day are ergogenic. However, the consensus was that acute supplementation with a nitrate dosage < 4 mmol/day is not ergogenic, which may indicate that this very low dosage is too small to appreciably alter NO bioavailability and impact downstream physiological function. It is noteworthy that the dosage of nitrate believed to be ergogenic will, in many cases, exceed the ADI of 3.7 mg/kg/day, and that many studies exploring the ergogenic effects of nitrate are also likely to have provided doses that surpass this threshold [2]. For example, a 70-kg individual would exceed their ADI of 259 mg/day nitrate by consuming just 4.2 mmol (~ 260 mg) of this compound. Moreover, many lighter individuals would be unable to consume enough nitrate to elicit an ergogenic effect (e.g. a minimum of 4 mmol, the amount deemed necessary for chronic nitrate supplementation to be ergogenic) without exceeding their ADI. Further discussion on the nitrate ADI is provided in the section on “*Safety and Toxicity*”.

*Acute versus chronic loading:* A consensus was not reached for whether chronic dietary nitrate supplementation is more likely to elicit ergogenic effects than acute supplementation, which may be related to both the ambiguity in available evidence and lack of studies directly comparing acute and chronic nitrate supplementation strategies. An early study by Vanhatalo et al. [11] reported a reduction in *V̇*O_2_ following both acute (2.5 h) and chronic (5 days and 15 days) dietary nitrate supplementation—an effect which might be expected to translate into improvements in exercise performance. Interestingly, 15 days of nitrate supplementation also increased ramp test peak power and the work rate at the gas exchange threshold. However, these effects were not apparent after shorter (2 h or 5 days) supplementation regimens. In contrast, Boorsma et al. [74] observed no effects of either acute (1 day) or chronic (8 days) dietary nitrate supplementation on submaximal *V̇*O_2_ or 1500-m running performance in elite distance runners. The difference in findings may be related to the high aerobic fitness of the participants in the study by Boorsma et al. [74], who had a mean *V̇*O_2peak_ of 80 ml/kg/min.

*Timing:* The expert panel agreed that acute dietary nitrate ingestion should occur 2–4 h before a performance test to elicit an ergogenic effect, and that consumption of dietary nitrate closer to (i.e. < 90 min) or further from (i.e. 6–8 h) the test is not ergogenic. As demonstrated by Wylie et al. [40], peak plasma nitrite concentration occurs ~ 2–3 h after dietary nitrate ingestion (which is ~ 1–2 h after peak plasma nitrate concentration, due to the requisite time for oral conversion of nitrate into nitrite). Conducting exercise during this time interval may maximise the chance of an ergogenic effect by ensuring the greatest circulating availability of substrates for NO production during exercise.

*Form:* The expert panel agreed that dietary nitrate is ergogenic when provided in the form of either beetroot juice (which has been used in the vast majority of studies) or nitrate salts (see [2]). In contrast, the expert panel felt that there was insufficient evidence for an ergogenic effect of dietary nitrate in the form of sport drinks, chewing gum, or gels, and a consensus was not reached for whether vegetables containing nitrate were ergogenic. The expert panel acknowledged that there was no reason why these sources of dietary nitrate could not be ergogenic, provided they contain a sufficient dose of nitrate. High doses of dietary nitrate can be achieved through the habitual diet [27, 75]. For example, the Dietary Approaches to Stop Hypertension (DASH) diet can contain up to ~ 20 mmol/day of nitrate [27]. However, this might not be practical for some athletes (e.g. due to food availability, dietary preferences or the perception that high intake of nitrate-rich foods such as vegetables pre-competition may cause gastrointestinal upset). Therefore, the use of specific high-nitrate supplements may be of value for athletes wishing to benefit from intake of this compound.

### Physiological Effects of Nitrate

Understanding the physiological effects that might underpin dietary nitrate-induced improvements in exercise performance has been of considerable interest in the literature. In the current study, it was agreed that chronic dietary nitrate supplementation reduces oxygen consumption during steady-state exercise. This effect, which was first established by Larsen and colleagues [76], has subsequently been confirmed by a number of independent research groups (for a review, see [77]), and has recently been verified via direct measurement of skeletal muscle oxygen uptake during exercise [78]. Interestingly, the expert panel were also in agreement that chronic dietary nitrate supplementation modulates the oral microbiota, an effect that has only recently been established. For example, Vanhatalo et al. [79] sequenced bacterial 16S rRNA genes after 10 days of supplementation with inorganic nitrate or a placebo and reported greater relative abundances of the genera *Rothia* and *Neisseria* and lower relative abundances of the genera *Prevotella* and *Veillonella* after nitrate versus placebo supplementation. Although these changes in the oral microbiome composition occurred alongside marked increases in NO biomarkers, it is important to note that the net oral nitrite production is influenced by a complex interplay between various nitrate- and nitrite-reducing microorganisms in the oral cavity. The influence of chronic dietary nitrate consumption on nitrate-reduction capacity of the oral microbial community in response to acute nitrate ingestion, and the impact of potential changes in relation to health and exercise performance, requires further exploration [71]. One recent study has shown positive associations between oral nitrate-reducing capacity and markers of aerobic fitness [80]. Regarding other physiological effects of nitrate (e.g. changes in mitochondrial efficiency, muscle calcium handling, microvascular blood flow, cognitive function), the expert panel agreed that there was either insufficient evidence or were unable to reach agreement.

### Safety and Toxicity

The expert panel agreed that acute dietary nitrate supplementation in dosages up to ~ 16 mmol/day does not increase risk of cancer, methaemoglobinaemia, hypotension or renal injury. Similarly, they agreed that chronic dietary nitrate supplementation in dosages up to ~ 16 mmol/day does not increase risk of methaemoglobinaemia or hypotension. However, given that few studies have explored the long-term health (or other) effects of nitrate, additional well-controlled longitudinal studies are required to support these views [71]. A consensus was not reached for whether chronic supplementation increases risk of cancer or renal injury, the former of which has been a subject of much debate in the extant literature [71]. As mentioned previously, dietary nitrate can be reduced into nitrite by bacteria in the oral cavity and when this nitrite is swallowed, a portion passes into the systemic circulation. However, the nitrite can also be acidified in the stomach to produce nitrous acid, which can potentially lead to the nitrosation of amines, forming carcinogenic *N*-nitrosamines [36]. Whether this translates into an increased risk of cancer is currently unclear, as was reflected in the panel’s conclusion. For example, a systematic review of observational studies suggests that nitrate does not increase site-specific cancer risk and may actually decrease the risk of gastric cancer [31]. Moreover, animal model studies have typically failed to show an increased risk of cancer with elevated nitrate intake [34]. By contrast, other observational studies have refuted these claims, reporting an increased risk of cancer with nitrate intake (e.g. [32, 33]). Based around an appraisal of the available evidence in 2010, IARC [34] concluded that there was ‘inadequate evidence in humans for the carcinogenicity of [dietary] nitrate’ whilst a 2016 review by WHO [35] concluded that ‘the weight of evidence does not clearly support an association between cancer and exposure to nitrate or nitrite per se’. Nevertheless, further research, especially longer-term randomized controlled trials in humans, is clearly required before firm conclusions can be drawn. From a holistic dietary perspective, the presence of antioxidants such as vitamin C and polyphenols in fruits and vegetables has been shown to prevent *N*-nitrosamine formation, which could mitigate risk of cancer with dietary nitrate consumed in these foods [81, 82]. In contrast, when nitrate/nitrite is consumed via processed meats (where these compounds are added as preservatives), there may be an increased chance of generating potentially carcinogenic *N*-nitrosamines if not consumed alongside sufficient vitamin C or other antioxidants [27, 83]. Therefore, those wishing to benefit from the potential ergogenic effects of dietary nitrate may be best advised to do so by consuming vegetable-derived nitrate supplements (e.g. beetroot juice), which were viewed as ergogenic by the expert panel and contain a range of antioxidants that may attenuate *N*-nitrosamine formation. Indeed, the expert panel agreed that there was no evidence for harmful effects of dietary nitrate when consumed via nitrate-rich foods and juices such as vegetables, salads and concentrated or non-concentrated beetroot juice. By contrast, they did not reach a consensus on potential adverse effects of nitrate salts, gels, drinks or gum.

The expert panel agreed that the nitrate ADI of 3.7 mg/kg/day is not appropriate based on current evidence. This is consistent with other reports in the literature calling for a revaluation of the nitrate ADI [27]. To help with the reformulation of these guidelines, long-duration, large-scale clinical trials in humans may be necessary to determine whether an upper limit to nitrate intake is required and, if so, what it should be and whether it depends upon the dietary nitrate source (e.g. drinking water vs. processed meats vs. vegetables).

When considering the potential health effects of nitrate under different environmental conditions, the expert panel agreed that both acute and chronic dietary nitrate does not increase risk of heat illness (e.g. hyperthermia) during exercise in the heat (> 30 °C). This is consistent with the findings from a series of studies by Kent et al. [84, 85], who showed no difference in core or skin temperature between dietary nitrate- and placebo-supplemented individuals exercising in the heat.

The expert panel were asked to comment on whether adverse health effects of dietary nitrate supplementation have been reported in specific populations. Following discussion in round three, two modifications were made to the original statements. Initially, ‘adverse events’ was modified to ‘serious adverse events’ to clarify that dietary nitrate supplementation has not been shown to lead to major negative health effects (e.g. death, hospitalisation, disability or incapacitation). Subsequently, it was agreed that serious adverse events have not been reported in *any* population following dietary nitrate supplementation, and the statement was consequently modified to encompass all individuals. Following these modifications, the expert panel agreed that serious adverse health effects of dietary nitrate supplementation have not been reported in any population.

### Ethos of Olympic Sport

Expert panel members were asked to consider whether dietary nitrate supplementation was consistent with the ethos of Olympic sport. Statements in this section were based on a previous Delphi study exploring the ergogenic effects of menthol [45], and were informed by the World Anti-Doping Agency (WADA) code [86]. Questions included (1) potential health risks of dietary nitrate supplementation; (2) whether supplementation is consistent with the spirit of the sport; and (3) whether supplementation confers an unfair advantage. A consensus was reached that acute dietary nitrate supplementation does not present an actual or potential health risk, whilst the panel were unable to reach consensus on whether chronic nitrate supplementation presents an actual or potential health risk. The expert panel agreed that both acute and chronic dietary nitrate supplementation does not violate the spirit of sport, nor does it provide an unfair advantage to those using it. Consistent with other legal nutritional ergogenic aids (e.g. caffeine, carbohydrate, creatine), dietary nitrate is found in a range of natural foods and is therefore widely available to individuals wishing to consume it. Importantly, this also means that it would be challenging to prohibit dietary nitrate consumption, unless high thresholds were imposed above and beyond typical intake levels.

### Quality of the Available Evidence

The expert panel identified that the quality of currently available evidence may be sub-optimal in several areas. For example, they agreed that most studies have used a performance test that is only partly indicative of the conditions that occur in real-world competitive sport. Indeed, most studies to date have included time-trial or time-to-exhaustion tests on a cycle ergometer or treadmill [2], which provide valuable insight into the effects of nitrate on exercise performance but may not mimic all of the technical, tactical and physiological demands placed on athletes during some types of competition [87]. Similarly, the expert panel agreed that most studies have an insufficient sample size and are likely to be underpowered. Moreover, most studies have only partly verified the nitrate content of the administered supplement, and the resulting biological response (e.g. via the collection of biological samples), and have only partly controlled for confounding variables that might influence the experimental results (e.g. mouthwash use, habitual diet, smoking status, pre-trial exercise levels, sleep, the testing environment). However, the expert panel identified that most studies were double-blind, randomized, controlled trials using a within-subject design, which should be considered a relative strength.

### Comparison with Recent Systematic Reviews and Meta-Analyses

Several high-quality systematic reviews and meta-analyses (e.g. [2, 88, 89]) have explored the ergogenic effects of dietary nitrate, the most recent of which was published by Senefeld et al. [2]. The findings from the current study did not always align with those of Senefeld et al. [2], and below, we highlight possible explanations for these discrepancies. We also discuss key strengths and weaknesses of systematic reviews/meta-analyses and the modified Delphi technique to help guide the interpretation of our findings.

Systematic reviews and meta-analyses are generally considered to be situated at the top of the hierarchy of evidence, and follow well-defined procedures to identify, appraise and synthesise the extant literature [90]. Strengths include the explicit, transparent methodology (which ensures reproducibility), the thorough search for evidence, the criterion-based selection of eligible studies, and the quantitative synthesis of research findings to provide a pooled estimate of overall effects [91]. However, systematic reviews are often limited by their narrow focus and adherence to rigid methods, which do not always allow for comprehensive coverage of the available evidence [91]. In contrast, the Delphi method has the benefit of being able to address a much broader set of questions (for example, we considered factors such as the safety and toxicity of nitrate and whether its use is consistent with the ethos of Olympic sport—areas not addressed in recent reviews [2, 88, 89]). In addition, systematic reviews are restricted by the limitations of the original studies, which serve as their raw ingredients [92]. In particular, the external validity/generalizability of findings from systematic reviews is determined by the external validity of the included studies, which may not always be optimal [92]. For example, studies exploring the effects of nitrate as an ergogenic aid have done so with tightly controlled participant groups (which may be narrow and not representative of all exercising individuals), specific exercise activities and supplementation protocols and data are typically derived from one-off tests [2]. This information may not translate particularly well to real-world scenarios that are typically characterized by a unique set of many moving parts. In contrast, with the Delphi technique, panel members can draw upon scientific evidence *and* practical experience gained from working with athletes and patients to inform their decision making. Whilst it is important to acknowledge the greater subjectivity inherent in this approach [44], it means that more nuanced interpretations can be made by drawing upon evidence from a variety of sources. Judgements may therefore be more ecologically valid and have greater utility for informing practice than an evaluation of effect sizes from published literature.

A particular discrepancy between the findings from Senefeld et al. [2] and this study, which demonstrates how these two methods may lead to different conclusions, was that, with their quantitative synthesis of the available literature, Senefeld et al. [2] demonstrated that dietary nitrate improves performance across a range of exercise modalities (including cycling, running and handgrip exercise, but not rowing or knee-extension exercise) and durations (especially events lasting < 1000 s or ~ 17 min). In contrast, our expert panel did not feel there was sufficient evidence to conclusively state that dietary nitrate is (or is not) ergogenic for any of the specified sporting/exercise situations. Our panel noted during the round three discussions that the ergogenic effects (or lack thereof) of dietary nitrate during any given activity could vary considerably depending upon the interaction between different methodological factors (see the section on ‘Modulators of the Ergogenic Effects of Dietary Nitrate’). Since the complete set of methodological permutations have not been fully elucidated, the expert panel felt unable to make a general statement about the ergogenic effects of dietary nitrate during specific activities based on the current evidence.

Ultimately, it is our view that systematic reviews/meta-analyses and the modified Delphi technique provide complementary information, one relying on a quantitative synthesis of scientific evidence, the other informed by expert judgement based around scientific and practical experience, which together provide valuable insight to help inform research and practice. Although we have highlighted some discrepancies in the findings from these two approaches, there is broad agreement on a number of key points (e.g. around optimal supplementation protocols and participant groups likely to benefit from nitrate), which provides confidence in the validity of these conclusions.

### Strengths and Limitations

A strength of this study is that our panel was composed of extensively published and cited research leaders and highly experienced practitioners, who are collectively responsible for many of the major developments in the area of dietary nitrate as an ergogenic aid. These individuals possessed a high level of knowledge, and direct research experience, of the majority of areas discussed. While this approach has some drawbacks, notably that some panel members may not have had sufficient knowledge to be able to respond to some specific statements, our data suggest that the panel members felt they had sufficient knowledge to contribute towards the appraisal of most statements. A further strength is that we recruited 12 individuals to serve as panel members, despite five to ten individuals typically being considered sufficient to form a consensus group [45, 47]. A third strength of this study is the diversity in our panel, which ensured a range of potentially different views. However, readers should be aware that results from the present study reflect the view of significant contributors to the field from a scientific and practitioner perspective, and may not necessarily be representative of the views of all researchers/practitioners in the field.

### Recommendations for Practice and Research

A summary of key practical recommendations from the current expert consensus are provided below.

*Population*

- Aerobic fitness should be considered when determining the potential ergogenic effects of dietary nitrate supplementation. Specifically, the current expert consensus is that acute and chronic nitrate supplementation is ergogenic for individuals with a *V̇*O_2peak_ < 45 ml/kg/min (low aerobic fitness), whilst chronic nitrate supplementation is ergogenic for individuals with a *V̇*O_2peak_ < 60 ml/kg/min (low and moderate aerobic fitness). The effects of nitrate appear to be diminished in highly trained individuals with a high aerobic fitness (e.g. *V̇*O_2peak_ > 60 ml/kg/min).

*Supplementation strategy*

- Athletes aiming to benefit from the ergogenic effects of nitrate may be advised to consume 8–16 mmol (e.g. 2–4 beetroot juice shots, depending upon supplement content) of dietary nitrate acutely or 4–16 mmol/day (e.g. 1–4 beetroot juice shots, depending upon supplement content) chronically (i.e. over several days or weeks).
- Dietary nitrate should be consumed 2–4 h before exercise to maximise the ergogenic response. Outside of this time interval the supplement may not be ergogenic.
- Current evidence suggests that dietary nitrate is ergogenic when consumed in the form of beetroot juice or nitrate salts. Other vehicles for nitrate delivery (e.g. whole vegetables) are likely to be ergogenic, providing they contain a sufficient nitrate dose. However, to maximise safety and performance, athletes may be best advised to consume nitrate in the form of beetroot juice or other vegetable-derived products (providing they contain sufficient nitrate alongside antioxidants) rather than via nitrate salts such as sodium or potassium nitrate.

*Physiological effects*

- Chronic dietary nitrate supplementation can reduce the oxygen cost of exercise, which may benefit athletes within a variety of sporting situations (e.g. prolonged endurance exercise, invasion games, combat sports.).
- Chronic dietary nitrate supplementation modulates the oral microbiota, the specific impact of which remains to be fully elucidated, but could have implications for health, as well as exercise performance.

*Safety and toxicity*

- The current expert consensus suggests that acute dietary nitrate supplementation in dosages up to ~ 16 mmol/day is likely to be safe and does not increase the risk of cancer, methaemoglobinaemia, hypotension or renal injury. Additional research is required to understand the health effects of chronic dietary nitrate consumption over prolonged periods (e.g. several months/years).
- Vegetables or vegetable juices may be the safest vehicle for consuming dietary nitrate, as they are rich in polyphenols and antioxidants, which can minimise the likelihood of forming potentially harmful *N*-nitrosamines, and there is no evidence for harmful effects following their consumption in dosages up to ~ 16 mmol/day.
- Dietary nitrate is unlikely to have adverse health effects in the heat, although further research is needed to understand the potential effects in other extreme environments (e.g. at altitude or in cold conditions).

*Ethos of Olympic sport*

- Consumption of dietary nitrate does not confer an unfair advantage and does not violate the spirit of sport.

### Future Research

Future research should aim to overcome key limitations present in the current body of evidence, including appropriate consideration for sample sizes, the translation of laboratory test results to real-world settings/conditions, lack of representation of female and older participants, and lack of independent verification of the nitrate content of administered supplements. Moreover, given that the expert panel often highlighted insufficient evidence to conclusively state whether dietary nitrate is/is not ergogenic for specific conditions, additional studies investigating the following are warranted:

- The performance effects of dietary nitrate in a range of scenarios that investigate ecologically valid sporting environments, including the following: events (e.g. different modes, intensities, durations); participants (e.g. different sexes, ages, fitness levels, physical disabilities); environmental conditions (e.g. heat, cold, altitude); dosing protocols (e.g. acute vs. chronic intake, intake during prolonged events); and special circumstances (e.g. repeated use in multi-events, co-ingestion with other supplements). Whilst it is acknowledged that these approaches may limit experimental control (i.e. introduce ‘noise’), they are likely to provide complementary insight into the ergogenic effects of dietary nitrate alongside the findings from well-controlled studies employing time-to-exhaustion or laboratory-based time-trial tests.
- Potential responders and non-responders to nitrate, and the consistency of the response within individuals. Future studies adopting repeated randomized crossover designs could be used to help identify consistent responders/non-responders [56, 93].
- The acceptability, pharmacokinetics and physiological/performance effects of other dietary nitrate sources besides beetroot juice (e.g. nitrate gels, chewing gum, vegetables).
- The impact of dietary nitrate on risk of altitude and cold-induced illnesses, which is not anticipated based around current knowledge, but may warrant direct exploration.
- The longer-term safety and toxicity of dietary nitrate, to rule out any potential adverse effects of chronically consuming this compound over several months/years.

### Conclusions

This study set out to produce an expert consensus on the use of dietary inorganic nitrate as an ergogenic aid using the modified Delphi technique, the findings from which could be of value to athletes, coaches, researchers and practitioners. Overall, a consensus was reached for 39 (20 yes, 19 no) out of 222 statements relating to the use of dietary nitrate as an ergogenic aid. The findings indicate that the effects of dietary nitrate appear to be diminished in individuals with a higher aerobic fitness (*V̇*O_2peak_ > 60 ml/kg/min), and therefore aerobic fitness should be taken into account when considering use of dietary nitrate as an ergogenic aid. It is recommended that athletes looking to benefit from dietary nitrate supplementation should consume 8–16 mmol nitrate acutely or 4–16 mmol/day nitrate chronically (with the final dose ingested 2–4 h pre-exercise) to maximise ergogenic effects, taking into consideration that, from a safety perspective, athletes may be best advised to increase their intake of nitrate via vegetables and vegetable juices. In addition, expert judgements concerning the safety and toxicity of dietary nitrate consumption are provided (specifically, that acute supplementation with ~ 16 mmol is likely to be safe, although further investigation into the safety of chronic supplementation is warranted), which could help athletes and coaches weigh up the potential advantages and disadvantages of nitrate supplementation. In addition, > 80% of the panel agreed that there was insufficient evidence to draw firm (yes/no) conclusions about the majority (134, 60.4%) of the appraised statements. This indicates a clear need for further research across many areas relating to the use of dietary nitrate as an ergogenic aid, the specifics of which have been highlighted in this paper.

## Supplementary Information

Below is the link to the electronic supplementary material.

Supplementary file1 (XLSX 186 KB)

## Declarations

### Availability of data and materials

Questionnaire response data are available in the electronic supplementary material.
